# Antimicrobial Resistance of Enteric *Salmonella* in Bangui, Central African Republic

**DOI:** 10.1155/2015/483974

**Published:** 2015-12-31

**Authors:** Christian Diamant Mossoro-Kpinde, Alexandre Manirakiza, Jean-Robert Mbecko, Pembé Misatou, Alain Le Faou, Thierry Frank

**Affiliations:** ^1^Laboratoire de l'Hôpital Maman Elisabeth Domitien, Bimbo, Central African Republic; ^2^Institut Pasteur de Bangui, P.O. Box 923, Bangui, Central African Republic; ^3^Faculté de Médecine and EA 3452, Cithefor, Faculté de Pharmacie, Université de Lorraine, Nancy, France

## Abstract

*Introduction*. The number of* Salmonella* isolated from clinical samples that are resistant to multiple antibiotics has increased worldwide. The aim of this study was to determine the prevalence of resistant* Salmonella enterica *isolated in Bangui.* Methods*. All enteric* Salmonella *strains isolated from patients in 2008 were identified and serotyped, and the phenotypes of resistance were determined by using the disk diffusion method. Nine resistance-associated genes,* bla*_*TEM*_,* bla*_*OXA*_,* bla*_*SHV*_,* tetA*,* aadA1*,* catA1*,* dhfrA1*,* sul I*, and* sul II*, were sought by genic amplification in seven* S*.*e*. Typhimurium strains.* Results*. The 94 strains isolated consisted of 47* S.e.* Typhimurium (50%), 21* S.e.* Stanleyville (22%), 18* S.e.* Enteritidis (19%), 4* S.e.* Dublin (4%), 4* S.e.* Hadar (4%), and 1* S.e.* Papuana (1%). Twenty-five (28%) were multiresistant, including 20 of the Typhimurium serovar (80%). Two main phenotypes of resistance were found: four antibiotics (56%) and to five antibiotics (40%). One* S.e.* Typhimurium isolate produced an extended-spectrum *β*-lactamase (ESBL). Only seven strains of* S.e.* Typhimurium could be amplified genically. Only phenotypic resistance to tetracycline and aminosides was found.* Conclusion*.* S.* Typhimurium is the predominant serovar of enteric* S. enterica *and is the most widely resistant. The search for resistance genes showed heterogeneity of the circulating strains.

## 1. Introduction

Salmonellosis is a common disease. The strains of* Salmonella enterica* are not responsible for typhoid fever but mainly for enteric infections; however, the commonest serovar, Typhimurium, may cause systemic infections, especially in immunocompromised patients and children with malnutrition, severe anemia, and malaria [1, 2]. Strains of* Salmonella* resistant to multiple antibiotics are being isolated more and more frequently. Thus, the commonly used antibiotics have become inefficient [3] and have had to be replaced by more expensive drugs [4]. The latest surveys conducted in the Central African Republic (CAR) showed a high prevalence of resistance to ampicillin and cotrimoxazole [5], which is increasing with time. To confirm this evolution, we determined the antibiotic resistance of strains of* S. enterica* isolated in 2008. Furthermore, we examined multiresistant strains of* S.* Typhimurium by genic amplification for the presence of nine of the commonest genes associated with antibiotic resistance.

## 2. Methods

The study was performed in the unit of Clinical Bacteriology and Antibioresistance of the Institut Pasteur de Bangui between July and December 2008. Ninety-four strains of* Salmonella* were isolated, mainly from stools (56%) and blood (36%); two strains were from urine and one was from cerebrospinal fluid.* Salmonella* were identified on the basis of biochemical characteristics (API 20^*E*^ strips, bioMérieux, Craponne, France), and the serovar was determined according to the Kauffmann-White scheme. The Antimicrobial drug susceptibility was determined by using the disk diffusion method (Bio-Rad, Marnes-la-Coquette, France) on Mueller-Hinton Agar (MHA) and interpreted according to the recommendations of the Comité de l'Antibiogramme de la Société Française de Microbiologie (CA-SFM) (http://www.sfm-microbiologie.org/). All isolates were tested for their susceptibility to antimicrobial agents routinely used in clinical practice for* Salmonella* infections in CAR. The antibiotics included ampicillin (25 *μ*g), amoxicillin (20 *μ*g), clavulanic acid (10 *μ*g), ticarcillin (75 *μ*g), cefalotin (30 *μ*g), cefotaxime (30 *μ*g), streptomycin (10 UI), gentamicin (15 *μ*g), nalidixic acid (30 *μ*g), ciprofloxacin (5 *μ*g), chloramphenicol (30 *μ*g), sulfonamides (200 *μ*g), and tetracycline (30 UI). If expanded-spectrum *β*-lactamase (ESBL) was found, additional antibiotics were tested (*β*-lactams and aminoglycosides). Strains were stored at −80°C in brain heart broth (Bio-Rad) with 20% glycerol.

Nine resistance-associated genes were sought by PCR with published techniques (Table 1) on six randomly selected strains and the ESBL-producing strain. After subculture on Mueller-Hinton Agar (Bio-Rad), DNA was extracted from the bacterial suspensions by thermolysis at 100°C in a water bath for 5 min, followed by rapid cooling at 20°C. After centrifugation (8855 ×g, 10 min), the supernatant was used for DNA amplification. PCR were run on a Gene Amp PCR System 9700 Thermocycler (Applied Biosystems, Saint-Aubin, France) as described previously [6, 7, 16]. Amplicon sizes were determined, from a molecular mass ladder (SmartLadder SF, EuroGentec, Angers, France) by gel electrophoresis in 2% ethidium bromide (Eurobio, Les Ulis, France) containing agarose (Invitrogen, Cergy-Pontoise, France) in Tris-acetate buffer at pH 8 and run at 100 V, 200 mA for 1 h. Results were read under UV. One negative (sterile distilled water) and three positive (*S.* Concord* 07-670*,* S.* Typhimurium* 02-8213*, and* Shigella dysenteriae* 1 CAR 10) controls were included.

## 3. Results

The 94 strains of* Salmonella* consisted of 47* S.e.* Typhimurium (50%), 21* S.e.* Stanleyville (22%), 18* S.e.* Enteritidis (19%), 4* S.e.* Dublin (4%), 3* S.e.* Hadar (4%), and 1* S.e.* Papuana (1%). Twenty-five strains (28%) were multiresistant: 20* S.e. Typhimurium* (80%), 3* S.e.* Enteritidis, 1* S.e.* Papuana, and 1* S.e.* Stanleyville. Among them, twenty-five isolates (28%) including* S.e.* Typhimurium (*n* = 20),* S.e.* Enteritidis (*n* = 3),* S.e.* Papuana (*n* = 1), and* S.e.* Stanleyville (*n* = 1) were multiresistant to antibiotics. The studied patients were constituted by 14 women and 11 men. They were between 1 month and 40 years of age and presented an average age of 11 years. The isolates were isolated from stool (*n* = 11), blood (*n* = 9), cerebrospinal fluid (*n* = 1), and urine (*n* = 1). All strains isolated from blood were represented by* S.e.* Typhimurium. Most of the strains were resistant to amoxicillin, ticarcillin, streptomycin, sulfonamides, and cotrimoxazole and less frequently to cefalotin (4%), ciprofloxacin (2%), gentamycin (3%), and nalidixic acid (4%). All the strains except one ESBL-producing* S.* Typhimurium strain (S1027072) were susceptible to cefoxitin. The ESBL-producing strain was resistant to amikacin, tobramycin, netilmicin, kanamycin, fosfomycin, ceftazidime, cefepime, aztreonam, and nitroxoline; it remained susceptible only to imipenem. Otherwise, two common resistance profiles were observed: 14 (34%) (12* S*. Typhimurium and 2* S*. Enteritidis) were resistant to four drugs (ampicillin, chloramphenicol, streptomycin, and sulfonamides) and 10 (24%) (7* S*. Typhimurium, 1* S*. Enteritidis, one* S.* Hadar, and one* S*. Papuana) were resistant to five drugs (ampicillin, chloramphenicol, streptomycin, sulfonamides, and tetracycline) (Table 2).

All strains carried the catA1 resistance gene, six contained* bla*_*TEM*_,* dhfrA1*, and *Sul II*, and five contained* sul I.* The genes* bla*_*OXA*_,* tetA*, and* aadA1* were found only once. The gene* bla*_*SHV*_ was not found, even in the ESBL-producing strain (Table 3).

## 4. Discussion

Three serovars of* S.e.* Typhimurium (*n* = 47),* S.e.* Stanleyville (21), and* S.e* Enteritidis (*n* = 18) constituted 96% of the isolated strains.* S.e.* Typhimurium and* S.e.* Enteritidis were the enteric* Salmonella* isolated most frequently [8, 9]. The surprisingly high prevalence of* S.* Stanleyville in Bangui may represent a locally circulating strain. The blood isolates were obtained mainly from AIDS patients [10–13].

*S.e.* Typhimurium was the* Salmonella* serovar that was most resistant to antibiotics. It is confirmed today that its multidrug-resistant strains have emerged in sub-Saharan Africa [17]. In Malawi, epidemics of multidrug-resistant invasive nontyphoidal* Salmonella* (defined as resistant to ampicillin, chloramphenicol, and cotrimoxazole) have been recorded [17]. The continuous increase in its resistance [14] will limit the therapeutic possibilities more and more, as exemplified by the emergence of an ESBL-producing strain (S1027072).

*Salmonella* resistant to four and five drugs are found widely in Africa. Although the prevalence in Bangui is high (58%), up to 82% of* Salmonella* isolates have been reported to be resistant [14, 15, 18]. Additionally, the prevalence of resistance to chloramphenicol, amoxicillin, and cotrimoxazole was high (72%), as reported in Lomé (Togo) [15], but is even higher in Taiwan (95%) [19]. These three cheap antibiotics used to be the first-line treatment for salmonellosis but can no longer be used. Third-generation cephalosporins and fluoroquinolones remain active against most strains of* Salmonella* in Bangui; in Asia, however, up to 54% of strains are resistant to these two antibiotics [20, 21]. Ciprofloxacin (or norfloxacin) is used as first-line treatment, except in children, for whom ceftriaxone is preferred [22]. Systematic use and self-medication with these antibiotics, which can be bought freely, raise concern that there might be a rapid increase in resistance [14, 21].

The use of tetracyclines and penicillin as growth promoters in animal husbandry is a factor in the increasing prevalence of resistance [23–25], but no information on this aspect is available in CAR.

The genes for which we searched only partly explain phenotypic resistance. There are many resistance genes, and one type of in vitro resistance may have several mechanisms. For example, aminoside resistance may be associated with three genes:* aphA* (aminoglycoside phosphotransferase),* aacC* (aminoglycoside acetyltransferases), and* aadA* and* aadB* (two variants of aminoglycoside adenyltransferases) [24], all of which are plasmid-encoded [25]. Antibiotic resistance may also be linked to other mechanisms, such as chromosomal mutations (modification of the ribosome structure, modification of the permeability of the cell wall, and presence of an efflux pump), which cannot be identified by genic amplification. Nevertheless, the presence of five genotypes among the six circulating* S.e.* Typhimurium strains resistant to four and five drugs indicates wide heterogeneity.

The ESBL strain with its seven resistance-associated genes differs from the other six; it is either imported or acquired a plasmid locally. The gene* CTX-M-15* has been described as the commonest in CAR and has been found in* E. coli* and* Klebsiella pneumoniae* strains [6]. As it was not present in the multiresistant* S.e.* Typhimurium strain, its origin remains unknown. It would be difficult to determine which EBSL is present, as no other isolate harboured this enzyme, and 230 ESBLs have been described so far [26]. The isolation of a multiresistant ESBL-producing strain in Bangui is worrying, as its spread would complicate patient care in view of the limited access in the country to the antibiotics to which such strains are susceptible [4, 20, 26]. A systematic study of isolated* S.e.* Typhimurium strains will be required to determine whether this strain is present in the country. For the moment, this appears unlikely, as no other isolate has been obtained.

The main limitation of our study is that data of the patient characteristics (age, sex, HIV status, malaria status, and malnutrition status) were not collected. Indeed, it would be essential to assess the impact of these characteristics with outcomes.

## 5. Conclusion

This preliminary study demonstrates a high prevalence of antibiotic-resistant* S.* Typhimurium and diverse associated genes in Bangui. Further studies will elucidate the epidemiology of the antibiotic resistance and make it possible to characterize the genes involved and the plasmids that carry them. In the absence of rational use of antibiotics in the country, continuous dissemination of resistant strains is likely. Systematic antibiograms should be performed for all isolated strains to follow the evolution of resistance and thus ensure effective treatment of infections, which are of particular concern for immunocompromised patients.

## Figures and Tables

**Table 1:** Primers used for detecting resistance-associated genes by PCR.

Gene	Enzymatic activity	Antibiotic targeted	Primers	References
*bla*_*TEM*_	Penicillinase TEM	Aminopenicillins, carboxypenicillins, and ureidopenicillins	OT3: 5′ ATGAGTATTCAACATTTCCG 3′ OT4: 5′ CCAATGCTTATTCAGTGAGG 3′	[6, 7]

*bla*_*OXA*_	Oxacillinase	Aminopenicillins, carboxypenicillins, ureidopenicillins, and Penicillin M	OXA F: 5′ ATGAAAACACAATACATATC 3′ OXA R: 5′ AATTTAGTGTGTTTAGAATG 3′	[8]

*bla*_*SHV*_	Beta-lactamase Sulfhydryl Variable (SHV)	All beta-lactams except carbapenems and cephamycins	OS5: 5′ TTATCTCCCTGTTAGCCACC 3′ OS6: 5′ GATTTGCTGATTTCGCTCGG 3′	[9]

*tetA*	Active efflux pump	Cyclines	TetA Lower: 5′ GCAGGCAGAGCAAGTAGAGG 3′ TetA Upper: 5′ GTTTCGGGTTCGGGATGGTC 3′	[6, 10, 11]

*catA1*	Chloramphenicol acetyl transferase	Chloramphenicol	CatA1-F: 5′ CGCCTGATGAATGCTCATCCG.3′ CatA1-R: 5′ CCTGCCACTCATCGCAGTAC 3′	[6, 12]

*aadA1*	Aminoglycoside adenyltransferase	Streptomycin	Aad-F: 5′ TATCAGAGGTAGTTGGCGTCAT 3′ Aad-R: 5′ GTTCCATAGCGTTAAGGTTTCATT 3′	[6, 13, 14]

*dhfrA1*	Dihydrofolate reductase (DHFR)	Trimethoprim	dhfrla-F: 5′ TGAAACTATCACTAATGGTA 3′ dhfrla-R: 5′ TTAACCCTTTTGCCAGTATTG 3′	[4, 15]

*sul I*	Dihydropteroate synthetase (DHPS)	Sulfonamides	Sul I-F: 5′ CGGCGTGGGCTACCTGAACC 3′ Sul I-B: 5′ GCCGATCGCGTGAAGTTCCG 3′	[6, 15]

*sul II *	Dihydropteroate synthetase (DHPS)	Sulfonamides	Sul II-F: 5′ GCGCTCAAGGCAGATGGCATT 3′ Sul II-B: 5′ GCGTTTGATACCGGCACCCGT 3′	[6, 15]

**Table 2:** Antibiotic resistance of *S. enterica* strains isolated in Bangui.

Antibiotic	Resistant strain													
	*S*. Typhimurium^*∗*^		*S. *Stanleyville		*S. *Enteritidis		*S. *Dublin		*S. *Hadar		*S. *Papuana		Total	
	Number	%	Number	%	Number	%	Number	%	Number	%	Number	%	Number	%
Ampicillin	20	43	1	5	3	17	1	25	2	67	1	100	29	31
Amoxicillin and clavulanic acid	8	17	0	0	1	6	0	0	1	33	0	0	11	12
Ticarcillin	20	43	1	5	3	17	1	25	1	33	1	100	28	30
Cefalotin	3	6	1	5	0	0	0	0	0	0	0	0	4	4
Cefoxitin	1	1	0	0	0	0	0	0	0	0	0	0	1	1
Cefotaxime	1	2	0	0	0	0	0	0	0	0	0	0	1	1
Streptomycin	20	43	1	5	3	17	0	0	0	0	1	100	25	27
Gentamicin	2	4	0	0	1	6	0	0	0	0	0	0	3	3
Nalidixic acid	3	6	0	0	1	6	0	0	0	0	0	0	4	4
Ciprofloxacin	2	4	0	0	0	0	0	0	0	0	0	0	2	2
Chloramphenicol	20	43	1	5	3	17	2	50	1	33	1	100	28	30
Cotrimoxazole	18	38	1	5	3	17	2	50	1	33	1	100	26	8
Sulfonamides	20	43	1	5	3	17	1	25	1	33	1	100	27	29
Tetracycline	7	15	1	5	2	11	0	0	0	0	1	100	11	12
Total	47		21		18		4		3		1		94	

^*∗*^Including one expanded-spectrum *β*-lactamase- (ESBL-) producing *S*. Typhimurium strain.

**Table 3:** Antibiotic resistance-associated genes in seven *S.e.* Typhimurium strains.

Gene	*TEM *	*bla*_*SHV*_	*tetA*	*bla*_*OXA*_	*aadA1*	*dhfrA1*	*catA1*	*sul I*	*sul II*
Control (+)	*S.* Concord		*S. dysenteriae* 1	*S.e. *Typhimurium		*S.* Concord	*S*.* dysenteriae *1		
	07-670		CAR 10	02-8213		07-670	CAR 10		

Strains resistant to ampicillin, chloramphenicol, streptomycin, and sulfonamides									
S0625010	+	−	−	−	−	+	+	+	+
S1107023	−	−	−	−	−	−	+	−	−
S0621014	+	−	−	−	−	+	+	+	+
S1028034	+	−	−	−	+	+	+	+	+

Strains resistant to ampicillin, chloramphenicol, streptomycin, sulfonamides, and tetracycline									
S0626101	+	−	−	−	−	+	+	−	+
S1003035	+	−	−	−	−	+	+	+	+

ESBL-producing *S.* Typhimurium strain									
S1027072	+	−	+	+	−	+	+	+	+

^+^Gene present; ^−^gene absent.
